# Approach to the asymptomatic patients with Brugada syndrome

**Published:** 2007-04-01

**Authors:** Masahiko Takagi, Hiroaki Tatsumi, Minoru Yoshiyama

**Affiliations:** Department of Internal Medicine and Cardiology, Osaka City University Graduate School of Medicine, 1-4-3 Asahimachi, Abeno-ku, Osaka 5458585, Japan

**Keywords:** Brugada syndrome, asymptomatic, sudden cardiac death, risk stratification

## Abstract

Brugada syndrome is an arrhythmogenic disease characterized by an ECG pattern of coved-type ST segment elevation in the right precordial leads and an increased risk of sudden cardiac death (SCD) as a result of polymorphic ventricular tachyarrhythmia or ventricular fibrillation (VF). Data from large patient studies and a meta-analysis of previous reports have shown that patients with a history of syncope or SCD and a spontaneous type 1 Brugada type ECG are at high risk for SCD. However, risk stratification of asymptomatic patients with Brugada type ECG is still a challenge. In particular, the use of electrophysiological study (EPS) for risk stratification remains controversial. Although some investigators have reported the possibility of use of EPS for distinguishing between high- and low-risk patients with Brugada type ECG, no precise predictor of risk for SCD in asymptomatic patients has yet been determined. The approach to treatment of these patients is thus still unclear. Large clinical prospective studies with uniform diagnostic criteria and protocols for EPS as well as extended follow-up periods of over ten years are required for prediction of SCD.

Since its description in 1992 [1], Brugada syndrome has been the focus of many clinical and research investigations as one of the leading causes of sudden cardiac death (SCD) in young adults. Recent large prospective studies have indicated that symptomatic patients with at least one episode of syncope or documented VF are at high risk of malignant ventricular tachyarrhythmias because of the high rate of recurrence of VF [2-4]. On the basis of this evidence, recommendations for treatment of this syndrome were described in the recently published consensus report [5]. The report recommends that symptomatic patients displaying type 1 (coved-type ST segment elevation ≥ 0.2 mV followed by a negative T wave) Brugada ECG (either spontaneously or after sodium channel blockade) who present with aborted SCD and related symptoms such as syncope, seizure, or nocturnal agonal respiration should undergo placement of an implantable cardioverter defibrillator (ICD) after non-cardiac causes have been ruled out. However, risk stratification of asymptomatic patients with Brugada type ECG is still a challenge.

## Risk stratification of asymptomatic patients with Brugada type ECG

Some reports have noted the natural history of asymptomatic patients with Brugada type ECG. Data from the largest patient series have shown that 8% of patients without previous cardiac arrest had SCD or documented ventricular fibrillation during a mean follow-up of 24 months [4]. In that study, patients with syncope and family members of patients with Brugada syndrome were included. The risk of these events was highest in patients who were men, had suffered syncope, had a spontaneously abnormal type 1 Brugada ECG, and had inducible ventricular tachyarrhythmias during electrophysiological study (EPS). Priori et al. found that 14% of patients with spontaneously abnormal Brugada type ECG (ST segment elevation ≥ 0.2 mV; whether type 1 ECG was observed was not specified) without previous syncope suffered cardiac arrest during a mean follow-up of 34 months, and that patients with spontaneously abnormal Brugada type ECG had relatively high risk of SCD [2]. In their study, family members of patients (35%) with Brugada syndrome were included. However, other studies reported a much lower risk of these events in asymptomatic patients [6-9]. Eckardt et al. found that only 0.8% of patients without previous cardiac arrest or syncope had SCD and documented ventricular fibrillation during a mean follow-up of 40 months [6]. They also reported that one of the significant predictors for risk of SCD was a spontaneously abnormal type 1 Brugada ECG. In the three Japanese studies, as well, although numbers of patients were relatively small and diagnostic criteria differed (not all patients had type 1 ECG), only 0.4 - 0.5% of sporadic patients without previous cardiac arrest or syncope had SCD per year [7-9].

These findings suggest that, although the risk of SCD in sporadic asymptomatic patients may be much lower, a spontaneously abnormal type 1 Brugada ECG might be a predictor for SCD in these patients.

Recent studies have suggested that combined noninvasive ECG markers may be helpful in risk stratification. Atarashi et al. reported the feasibility of risk stratification using the width of the S wave and ST segment elevation magnitude in the right precordial leads on surface ECG [10]. Some reports noted that signal-averaged ECG (SAECG) has been useful for identifying patients at high risk [11,12]. Other studies have suggested that circadian and daily fluctuations in ECG and SAECG could be useful for distinguishing between high- and low-risk patients [13-15]. The value of these markers in combination remains to be tested in a large prospective study.

## Role of electrophysiological study in risk stratification

The predictive value of inducibility of sustained ventricular arrhythmias during EPS is still controversial. Although Brugada et al. have supported the value of EPS based on the results for the largest series of patients [4], two other large studies have not confirmed their findings [2,6]. Recently, Gehi et al. performed a meta-analysis of prognostic studies of patients with Brugada syndrome [16]. The results of their meta-analysis did not support the value of EPS as a predictor of cardiac events. The heterogeneity found in the value of EPS for risk stratification may be due to methodological differences in stimulation protocols and/or criteria for positive EPS [2,6]. A standard methodology is needed to determine the value of EPS for risk stratification in patients with Brugada syndrome [17].

## Therapeutic approach to asymptomatic patients with Brugada type ECG

Currently, implantation of an ICD is the only effective treatment for this syndrome. However, various other treatments have been attempted to prevent SCD. Quinidine has been shown to be effective for prevention of SCD [18-20]. Tedisamil and isoproterenol have been reported to normalize the ST segment elevation in patients with this syndrome [21,22]. The phosphodiesterase III inhibitor, cilostazol, has also recently been reported to be useful [23]. However, the long-term efficacy of these treatments in preventing SCD is still unclear.

For symptomatic patients with type 1 Brugada ECG (either spontaneously or after sodium channel blockade), ICD implantation is recommended in the report of the second consensus conference [5]. With regard to treatment of asymptomatic patients, the indications for use of ICD remain controversial. The report of the second consensus conference recommends ICD implantation for asymptomatic patients with a family history of SCD, type 1 Brugada ECG (either spontaneously or after sodium channel blockade), and inducible ventricular tachyarrhythmias during EPS. However, the meta-analysis of prognostic studies of patients with Brugada syndrome by Gehi et al. did not support the value of EPS and family history of SCD as predictors of cardiac events. For asymptomatic patients with spontaneous type 1 Brugada ECG without family history of SCD, the indications for treatment remain unclear, since the value of EPS is still controversial, as noted above. Asymptomatic patients with type 1 Brugada ECG after sodium channel blockade and without family history of SCD appear to be at low risk for SCD [2].  Treatment is currently not recommended for these patients.

There has been some lack of uniformity in the diagnostic criteria and protocols for EPS, and follow-up periods have been limited. Large clinical prospective studies with uniform diagnostic criteria and protocols for EPS as well as extended follow-up periods of over ten years are required for prediction risk of SCD in asymptomatic patients with Brugada syndrome.
